# The Diverse Search for Synthetic, Semisynthetic and Natural Product Antibiotics From the 1940s and Up to 1960 Exemplified by a Small Pharmaceutical Player

**DOI:** 10.3389/fmicb.2020.00976

**Published:** 2020-06-12

**Authors:** Jørgen J. Leisner

**Affiliations:** Department of Veterinary and Animal Sciences, Faculty of Health and Medical Sciences, University of Copenhagen, Copenhagen, Denmark

**Keywords:** antibiotic, antimicrobial compound, LEO Pharma, screening, tuberculosis

## Abstract

The 1940s and 1950s witnessed a diverse search for not just natural product antibiotics but also for synthetic and semisynthetic compounds. This review revisits this epoch, using the research by a Danish pharmaceutical company, LEO Pharma, as an example. LEO adopted a strategy searching for synthetic antibiotics toward specific bacterial pathogens, in particular *Mycobacterium tuberculosis*, leading to the discovery of a new derivative of a known drug. Work on penicillin during and after WWII lead to the development of associated salts/esters and a search for new natural product antibiotics. This led initially to no new, marketable compounds, but concluded with the serendipitous discovery of fusidic acid, an antibiotic used to treat infections by *Staphylococcus aureus*, in 1960. The discovery process included contemporary approaches such as open innovation; targeting specific pathogens and/or specific organs in the patient; examining the effects of antimicrobial compounds on bacterial virulence as well as on antibiotic-resistant variants, and searching for antibiotic producers among microorganisms not previously well explored. These activities were promoted by the collaboration with a renowned Danish clinical microbiologist, K. A. Jensen, as well as company expertise in fermentation technologies, chemical synthesis and purification of bioactive compounds from organic materials.

## Introduction

This review covers the 1940s and 1950s that witnessed the golden age of discoveries of naturally occurring antibiotics (Table 1), with the American pharmaceutical industry as a major player (Anon, 1958; McGraw, 1976; Temin, 1979; Greenwood, 2008). The American natural product antibiotic manufacturers at the time included Abbott, American Cyanamid (Lederle), American Home Products, Bristol-Myers, Commercial Solvents Corporation, Eli Lilly, Merck, Olin Mathieson, Parke-Davis, Penick, Pfizer, and Upjohn (Anon, 1958). They represented a mixture of holding-operating and pharmaceutical companies, all with a diversified line of pharmaceutical products. Large-scale empirical screening programs for natural product antibiotics constituted an important activity for several of these companies at the time, but for some of them empirical searches were a temporary aberration from research based on synthetic organic chemistry (Daemmrich, 2009).

**TABLE 1:** Discovery milestones of synthetic and natural product antibiotics 1943–1960, with indication of application toward tuberculosis.

**Product class/compound (example)**	**Application**	**Discovery^a^**
Para-aminosalicylic acid (PAS)	Tuberculosis	1943 (s, i/p)
Aminoglycosides (streptomycin)	Tuberculosis	1943 (n, p)
Cyclopeptides (bacitracin)		1943 (n, p)
Thiosemicarbazone (thiacetazone)	Tuberculosis	1944 (s, i)
Nitrofurans (nitrofurantoin)		1944 (s, i)
Cephalosporins		1945 (n, p)
Chloramphenicols (chloramphenicol)		1945 (n, i)
Tetracyclines (chlortetracycline)		1945 (n, i)
Cyclopeptides (polymyxins)		1946 (n, i)
Diaminopyrimidines (trimethoprim)		1947/1956^b^ (s, i)
Macrolides (erythromycin)		1942/1950^b^ (n, i/p)
Isoniazid	Tuberculosis	Around 1950 (s, i)
Pleurimutilin		1951 (n, p)
Nicotinamide derivative (Pyrazinamide)	Tuberculosis	1952 (s, i)
Streptogramins		1953 (n, i)
Glycopeptides (vancomycin)		1953 (n, i)
Nitroimidazoles (metronidazole)		1953/1957^b^ (n/s, i/p)
Cycloserine	Tuberculosis	1954 (n, i)
Aminocoumarin (novobiocin)		1955 (n, i)
Rifamycins	Tuberculosis	1957 (n, i)
**Fusidic acid**		**1960 (n, i)**

*Letters in bold indicate discovery by LEO. ^*a*^Inconsistencies were noted among sources regarding years recorded for discoveries. Data are primarily from Greenwood, 2008 but also from Kavanagh et al., 1951; McGraw, 1976; Silver, 2011; Lewis, 2013; and Genilloud, 2014. n, natural product antibiotic; s, synthetic antibiotic; i, first compound was discovered in an industrial laboratory; p, first compound was discovered in a laboratory belonging to a public institution. ^*b*^Year indicate discovery of compound listed as example.*

The discovery approach for naturally occurring antibiotics was simple and consisted of screening primarily soil microorganisms on a range of agar media for antagonism toward selected target organisms: the Waksman platform (Aminov, 2010; Lewis, 2013; Wright, 2014; Katz and Baltz, 2016), although screenings with liquid cultures were preferred by Eli Lilly from the early 1950s (McGraw, 1976). Such empirical screenings involved compounds whose molecular nature was unknown at the time of discovery, and their subsequent purification, structural characterization and studies of the mode of action were frequently a challenging process. Deciding whether a screening had exhausted the possibilities, likely depended on the moment when the cost of failures and false leads exceeded the limit of economical or other constraints set by the company. Failures constituted a common experience only now and then punctuated by the rare discovery that lead to the marketing of a new product. One publication from 1950 stated that “…the principal objective is to differentiate between already known antibiotics, which are of but little interest, and new antibiotics which are of little interest, and new antibiotics which might find usefulness in chemotherapy. In our experience most of the antibiotic-producing soil organisms which survive the initial bacteriological screening are eliminated here…after screening several hundred thousand soil microorganisms, one is unusually fortunate if one or two organisms continue to be of interest” (Kane et al., 1950).

**FIGURE 1:**
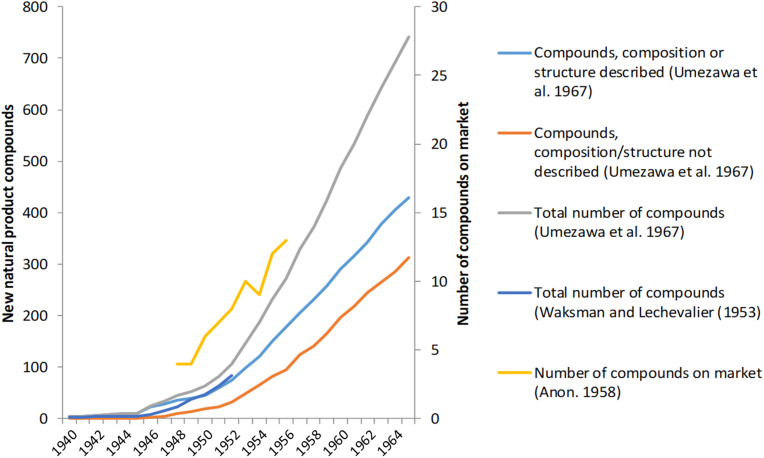
Discoveries of new natural product antibiotics produced by actinomycetes 1940–1965 (data from Waksman and Lechevalier, 1953 and Umezawa et al., 1967) and numbers of actinomycetes antibiotics available on the American market during the late 1940s-early 1950s (data from Anon, 1958).

Even so, an optimistic approach frequently prevailed: “Everywhere the searchers say: ‘If it can happen once, surely it can happen again”’ (Raper, 1952). Greenwood (2008) also mentions “the intense excitement of the time” (p. 225), and R. G. Benedict who was involved in an antibiotic discovery program at Northern Regional Research Laboratory at Illinois, noted that “the discovery of these agents has stimulated the hope that other antagonistic strains, yet undiscovered among the actinomycetes, may provide additional useful tools” (Benedict, 1953). Figure 1 illustrates the high degree of discoveries up to 1965 of novel compounds within this area as well as the high proportion of cases with described chemical compositions or structures. From the 1960s, it became apparent that the possibilities for finding new marketable compounds by the Waksman platform were exhausted. The low hanging fruits were gone (Quinn, 2009; Greenwood, 2008; Silver, 2011; Lewis, 2013, 2015; Wright, 2014). Empirical screenings for such compounds still have relevance today, as many of the initial shortcomings have been addressed. These shortcomings include risk of rediscovery, antibiotic resistance, lack of methodology for cultivating antibiotic-producing bacteria, maintaining their stability for antibiotic production, complex chemical structures, and labilities of compounds that required specialized equipment and expertise for purification and characterization (Peláez, 2006; Aminov, 2010,2017; Silver, 2011; Lewis, 2013,2015; Genilloud, 2014; Spellberg, 2014; Wright, 2014; Katz and Baltz, 2016; Abouelhassan et al., 2019). Regarding antibiotic resistance the range of times between introduction and the first observation of resistance varied from almost immediately, as was the case for penicillinase-resistant *Staphylococcus aureus* while in other cases such as vancomycin resistance in enterococci it took several years (Silver, 2011). Interestingly, the presence of antibiotic-resistant *S. aureus* in hospital environments was a driver for searching for new antibiotics already in the early 1950s (McGraw, 1976; Gradmann, 2016). This issue became even more important later on with the introduction in the 1960s of semisynthetic penicillins not susceptible to penicillinases as an example (Greenwood, 2008; Aminov, 2010; Gradmann, 2016).

During the 1930s and early 1940s, the pharmaceutical industry synthesized sulfonamides and sulfones to obtain compounds with improved antimicrobial, pharmacological and toxicological characteristics. These examples on synthetic antibiotics (organic structures synthesized in the laboratory) included more than 5000 sulfonamide derivatives by the end of WWII (Greenwood, 2008). After WWII, the pharmaceutical industry continued research on synthetic compounds with anti-tuberculosis drugs constituting a substantial part of these activities. This bacterium is difficult to treat due to the permeability barrier presented by its cell envelope, and the nature of the complex lesions in infected patients (Tanner et al., 2018). Further, tuberculosis requires prolonged multi-drug treatment to prevent treatment failure due to the emergence of resistance. This resistance was in the 1940s and 1950s to a higher degree linked to individual patients rather than a result of being spread within populations as has been the case for multi-drug resistant strains during the 1980s and 1990s (Keshavjee and Farmer, 2012; Gradmann, 2016, Fox et al., 2017). As for penicillin-resistant *S. aureus* variants, this situation led to searches during the 1940s and 1950s for new drugs that resulted in discoveries of synthetic compounds targeting the tuberculosis bacterium (Table 1). The introduction of synthetic compounds against other bacterial pathogens also took place during this time (Table 1; Greenwood, 2008; Wright et al., 2014). In contrast to natural product antibiotics, the known chemical structures of synthetic antibiotics made it possible in some cases to predict, whether a specific synthetic approach would lead to the desired result. One example is a research summary of work done from 1943 to 1953 at the company that constitutes the focus of this study, LEO Pharma. It was noted how many synthetic compounds within a given category, it would be reasonable to synthesize to ensure with an acceptable degree of accuracy that no active compounds were left untested^1^.

Chemical synthesis of naturally occurring antibiotics turned, on the other hand, out to be too difficult or expensive in most cases with chloramphenicol as the prominent exception. Instead, there are examples early on of the development of derivatives of natural fermentation products (semisynthetic antibiotics) such as dihydrostreptomycin and tetracycline. Overall, the value of semi-synthetic antibiotics was recognized from the work on β-lactams at the end of the 1950s.

I illustrate the work done on antibiotics in the 1940s and 1950s by examining one particular example: the research done by the small Danish company LEO Pharma. This company manufactured at that time several different drugs, many of them belonging to other categories than antibiotics. The company was active in research and development of natural product, synthetic and semisynthetic antibiotics frequently in close collaboration with an internationally renowned medical microbiologist (K. A. Jensen) with strategies depending to a large degree on target species and pharmaceutic issues. K. A. Jensen was involved in the first result from the empirical search for natural product antibiotics: the re-isolation of penicillin during WWII. Subsequent screening for naturally-occurring compounds with its failures and ultimate success was more a company activity that only initially involved another external collaborator in a supportive role. The work by LEO focused also on challenges such as antibiotic resistance and targeting virulence properties of bacterial targets, a potentially important strategy to counteract selection for resistance. Finally, it is of interest how LEO supported the innovation pipeline, by applying the open-source approach and searching for new natural product antibiotics among poorly investigated microorganisms.

The material for this review comes from different sources. The scientific literature offered substantial information, including quantitative data for discovery rates for actinomycetes antibiotics 1940–1965 obtained from Umezawa et al. (1967) and Waksman and Lechevalier (1953) (see Supplementary Appendix S1 for details). I have preferred to use the non-taxonomic term actinomycetes (covering the important genus *Streptomyces*) due to it has been used in a number of sources I quote. The primary source material on antibiotic research from 1940 to 1960 is in the archive of LEO Pharma Historical Archives and Museum. My manual search of this archive, across numerous visits, was essential to the completion of this review. This archive supplied meeting memos and summaries, letters and research reports, timesheets for chemists 1946–1959, two hand-written laboratory notebooks for the period from 1951 to 1956, orders for target strains for screenings and lists outlining the contemporary organization of the company into research units. Finally, I interviewed a researcher who was involved in the discovery of fusidic acid at LEO.

## Overview of LEO Pharma’s Research and Product Portfolio

The purchase of a pharmacy store in 1908 marks the beginning of the company. In 1909, LEO started production of a yoghurt preparation, and marketed an aspirin product soon thereafter. Then, in 1917 was launched the first Danish export drug, Digisolvin, a standardized Digitalis-based product. Hormones became a part of the product portfolio from the 1920s (Schrøder, 2005; Schrøder et al., 2008). During the 1940s and 1950s, the company maintained a broad portfolio of products of which several were generics (Loldrup, 2014)^2^. Examples include anesthetics (e.g., Citodan 1939–1956, Leostesin 1954–1988), diuretics (e.g., Diuregan 1929–1957, Diural 1937–1945, Rontyl 1958–1996) and hormones (e.g., Delcortol 1957–1996, Heparin from 1940, Solvisat 1957–1976, Testex 1937–1973) in addition to antibiotics. The company also worked on several smaller development projects and product categories. The majority of drugs were synthetic compounds whereas the hormones constituted natural compounds extracted from animal tissues or human urine. The know-how in natural product chemistry obtained by working with hormones proved important concerning subsequent purification of natural product antibiotics.

**FIGURE 2:**
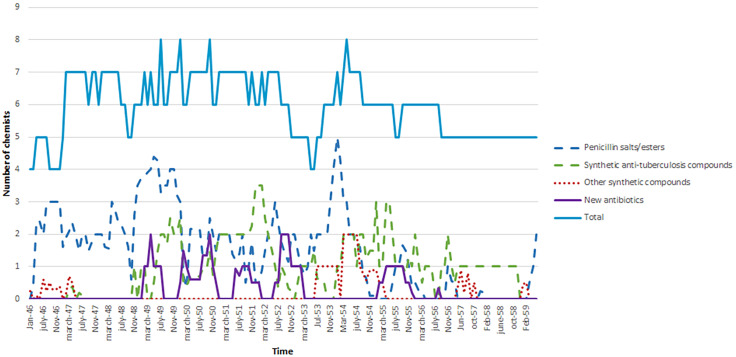
Overall numbers of chemists at the Chemical Research Laboratory A and the Hormone Factory and specific numbers of chemists working on various categories of antibiotics at LEO from 1946 to 1959. In some cases, the workload by the individual chemist is not expressed as a whole number. This use of a non-whole number is due to their time allocation among different projects, only some of which was concerning antibiotics. New antibiotics = New natural product compounds.

**FIGURE 3:**
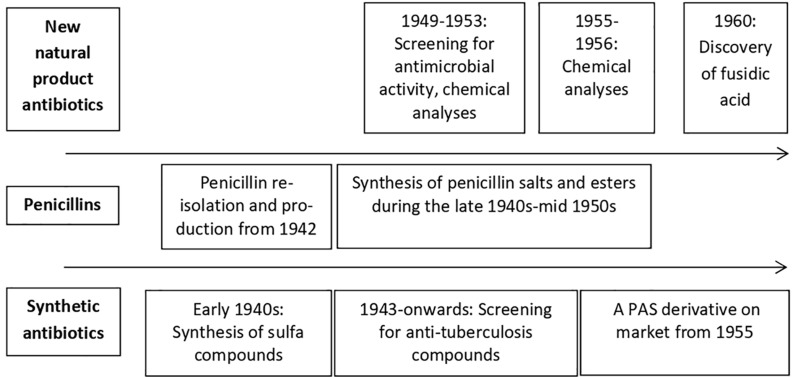
Timeline for the major activities within the antibiotic screening programs at LEO 1940–1960.

Work done by the chemical laboratory of the company from 1943 to 1953 indicated an increase in ambitions as activities changed from mostly working on drugs new to the Danish market, but well known from the literature, to rely on original research on a wide range of new drugs including antibiotics. In 1953 only one technician and one chemist out of six and seven, respectively, in that laboratory worked on the synthesis of a known compound with known effects while the remaining all carried out original work^3^. From 1946 to 1959, around 50% of research time was devoted to antimicrobial compounds (Table 2). This research focused on penicillin salts and esters and synthetic anti-tuberculosis compounds whereas research on new, unknown natural product antibiotics only amounted to minor amounts of research time (Table 2 and Figure 2). To this effort, however, should be added time spent on screening and characterizing antibiotic-producing microbial cultures by other departments and laboratories within the company than those that employed the chemists and for which there are no quantitative data. Figure 3 presents timelines for work by the company on the different categories of antibiotics. The company was active in the field of antibiotics until the 1980s. Attention then turned toward dermatological products that have remained the core area of business for the company up to this day.

**TABLE 2:** Main programs and collaborators in antibiotic research programs at LEO 1946–1960.

**Research program**	**Collaborator**	**% time of work by chemists^a^**	**Duration**	**New lead compounds**	**New derivatives**
Penicillin salts/esters	K. A. Jensen	26%	1946–1959	No	Yes
Synthetic antibiotics towards tuberculosis	K. A. Jensen	16%	1947–1958	No	Yes
Other synthetic antibiotics	K. A. Jensen	3%	see Figure 2	No	No
New natural product antibiotics	H. L. Jensen	5%	1949–1956	No	No
Fusidic acid		No data	1960	Yes	No
In total		50%	1946–1960	Yes	Yes

*^*a*^Data were extracted from the monthly overviews on which subjects the chemists were working on. A total of 50% of the research time by chemists at the Chemical Research Laboratory A (Chemical synthesis) and the Hormone Factory was spent on antibiotics. The remaining time was used on a variety of compounds targeting other conditions than bacterial infectious diseases. The numbers do not give a precise impression on the overall work dedicated by the company to antibiotics as work in other sections is not quantified. Research on penicillin manufacturing 1946–1949 is included in the percentage for penicillin salts/esters.*

## Work at LEO on Synthetic and Semisynthetic Antibiotics 1940–1960

The company collaborated from the 1930s with the medical bacteriologist, Kai Adolf (KA) Jensen (1971) on synthesis and testing of chemotherapeutics toward tuberculosis. Jensen was an internationally renowned researcher on this disease in addition to the chemotherapeutical application of antimicrobial compounds. He was associated with the State Serum Institute in Copenhagen until 1940 when he became a professor at the Department of General Pathology, University of Copenhagen. One of his research programs focused on selecting comparatively simple synthetic compounds on the basis of a literature search and subsequent synthesis of derivatives on a rational basis to examine their chemotherapeutic effect toward tuberculosis^4^. This collaboration continued during the 1940s and 1950s with Jensen in multiple roles as consultant, performing tests of the antimicrobial effects of compounds on laboratory animals and in some cases also organizing clinical trials (Jensen, 1971, 2002; Høiby, 2000, 2016). An example included a p-aminosalicylic acid (PAS) derivative sent in 1954 to some Danish hospitals and tuberculosis sanatoriums^5^.

The situation after WWII with tuberculosis on the rise again in Europe, contributed to the urgency of the efforts as was also the case for other pharmaceutical companies such as Bayer in Germany (Gradmann, 2016)^6^. Developing new antimicrobial compounds toward this disease constituted an important activity at the company until the end of the 1950s (Table 2 and Figures 2, 3)^7^. The company observed in 1949 that even after the introduction of streptomycin and PAS “a really good chemotherapeutic agent was still lacking against tuberculosis”^8^. The introduction of new methods in the form of improved growth media and new experimental animals allowed more rapid test procedures. Further, a specific method, the “object-glass method,” probably as described by Espersen (1949), developed in Denmark and employed by LEO allowed an easy way to determine whether a compound killed the tuberculosis bacteria or only inhibited growth and, importantly, whether it gave occasion for selection of resistant variants. In conclusion, a combination of such methods gave LEO “a real chance to direct the investigations toward a successful result”^9^. The objective was to examine all organic compounds in the company chemical collection and to synthesize derivatives of various lead compounds^10^. This amounted to around 500 compounds in 1956, resulting in the discovery of a new p-aminosalicylic acid phenyl ester (“Tebamin”; see below)^11^.

Specific examples of work on anti-tuberculosis compounds included in 1943–1944 sulfonamides and sulfones and from 1950 to 1953 derivatives of known lead compounds including 53 derivatives of PAS (discovered in 1943, Table 1), 62 derivatives of thiacetazone (discovered in 1944, Table 1) as well as several isoniazid derivatives (discovered around 1950, Table 1)^12^. Tuberculosis requires a long period of antibiotic treatment, which increases the probability of selection of resistant variants. This aspect had the attention of the scientific community already in the late 1940s (Jain et al., 2008; Gradmann, 2016). LEO tested some isoniazid derivatives toward isoniazid-resistant variants of *Mycobacterium tuberculosis* showing the interest of LEO on the issue of antimicrobial resistance^13^. LEO also researched whether isoniazid and a related compound, nicotinamide diminished the pathogenic potential of various bacterial target organisms including *Klebsiella pneumoniae*, *Salmonella*, *Shigella* and staphylococci in addition to *Mycobacterium tuberculosis*^14^. This topic has gained recent attention, due to the hypothesis that such an anti-virulence effect might not as rapidly lead to antibiotic resistance as is the result of bacteriostatic or bactericidal effects (Rasko and Sperandio, 2010; Wright, 2014; Theuretzbacher and Piddock, 2019).

The company also aimed at synthesizing derivatives of anti-tuberculosis compounds with an improved lung affinity, however, these compounds showed too high toxicity^15^. Another line of research, suggested by K. A. Jensen, focused on the effects of serum binding on activity. Hydrazides and PAS derivatives were of particular interest^16^. A PAS-derivative, p-aminosalicylic acid phenyl ester (“Tebamin”) was developed with a less irritating effect on patients than PAS and therefore suitable for the long duration of chemotherapeutic treatment of tuberculosis (Frederiksen et al., 1957; Tørning et al., 1958; Kingston, 2000; Greenwood, 2008; Jain et al., 2008). This compound was on the market from 1955 and up to 1973 and constituted the most successful outcome of LEOs work on anti-tuberculosis compounds (Jensen, 1971; Loldrup, 2014)^17^.

Chemical synthesis of antimicrobial compounds, many generics, targeting other infectious diseases than tuberculosis took place during the 1940s and 1950s. Three anti-syphilitic agents were synthesized, and one of these was on the market from 1941 to 1953 (Loldrup, 2014)^18^. The antifungal effect of more than 200 compounds, including 50 new synthetic compounds were examined between 1943 and 1953 but not resulting in new lead compounds^19^. Sulfonamides constituted an area of much interest in the early 1940s, with the synthesis of at least 30–40 derivatives up to 1944. Some of these included known compounds (e.g., N′-dimethylacrylsulfanilamide) whereas others were new or with unknown antimicrobial activities (e.g., sulfanilic acid hydrazide). The compounds were, in collaboration with K. A. Jensen, tested toward a panel of bacterial targets including anaerobic bacteria, enterococci, *E. coli*, pneumococci, streptococci and “proteus bacteria.” Some of the results appeared promising at first, but no lead compounds were found^20^.

LEO did some limited research on semisynthetic derivatives of chlortetracycline during 1953 and 1954^21^. The archives do not contain information on the objectives nor the outcome of this research. Most likely, it was inspired, by the launch by Pfizer in 1953 of tetracycline, a semisynthetic product obtained by cleaving the carbon-chlorine bond of the natural compound chlortetracycline (Greenwood, 2008; Wright et al., 2014). Although a natural version of tetracycline was also obtained around that time, the observation that it could be made as a derivative of chlortetracycline demonstrated that natural compounds could be perceived as starting points for the semisynthetic discovery process (Wright et al., 2014). LEO pursued this line of research further at the end of the 1950s after the discovery by the United Kingdom Company, Beecham, of a new way to produce semisynthetic penicillin derivatives from 6-aminopenicillanic acid. LEO subsequently introduced the semisynthetic mecillinam in 1972 (Greenwood, 2008). Ironically, this research also initially led to the serendipitous discovery of fusidic acid, a natural product antibiotic that became a highly successful product for LEO from 1960 and onward.

## The Search for Natural Product Antibiotics at LEO During the 1940S and Early 1950S

LEO’s involvement in the Danish efforts to develop an independent production of penicillin during and after WWII added research on natural product antibiotics to their activities. The penicillin story started with the owner of LEO, Knud Abildgaard (1901–1986) asking K. A. Jensen in 1942 whether he would be interested in extending their collaboration to include this topic. Jensen isolated the same year a penicillin-producing mold from the air in his laboratory, similar to Fleming’s discovery in 1928. LEO collaborated with K. A. Jensen on developing the manufacturing process that went on in secrecy due to the German occupation of Denmark during WWII (Jensen, 1971, 2002; Gotfredsen, 1991; Schrøder et al., 2008; Cozzoli, 2014; Tjørnelund, 2016). The company undertook also screenings to find penicillin-producing mold cultures^22^. Early on, it was clear that it was not easy to synthesize penicillin G and the company took a fermentative, not a chemical approach to manufacturing (Tjørnelund, 2016). Producing penicillin in bulk by fermentation became a success story for LEO that gave important know-how for optimizing cultivation techniques for the production of antibiotics and introduced the company as a pharmaceutical player internationally.

During the late 1940s and 1950s there was an interest in developing salts or esters of penicillin to overcome problems of poor absorption and rapid excretion as well as to improve organ affinity, such as for the lungs, whereas the activity of the compound was unaltered (Greenwood, 2008). Fifty different penicillin esters had been tested in collaboration with K. A. Jensen by 1951, increasing to at least 75 different compounds in 1954 (Table 2 and Figures 2, 3)^23^. One outcome was Leocillin (Benzylpenicillin, β-diethylaminoethyl ester hydrochloride) with a high lung affinity that found application as a veterinary product (Jensen et al., 1951; Schrøder, 2005). This compound was an inspiration to LEO to search for anti-tuberculosis compounds with similar affinities as mentioned previously^24^. Further work also resulted in the successful marketing in 1956 of a calcium salt of a variant of penicillin, V-penicillin in addition to an ampicillin ester in 1967 and an esterified combination of ampicillin and a beta-lactamase inhibitor in 1979 (Gotfredsen, 1999a, b; Schrøder, 2005).

In contrast to the work on penicillin, K. A. Jensen was not involved in the natural product antibiotic screening program. Production of additional antibiotics, either known compounds on license such as streptomycin or entirely new compounds, would be an economic incentive to keep the penicillin production plant at LEO busy^25^. The company was also aware of the need to find new broad-spectrum chemotherapeutic compounds with activity toward bacterial pathogens resistant to penicillin, streptomycin and sulphonamides^26^. Most likely, *M. tuberculosis* and *S. aureus* were among the pathogens that had the attention in that regard. A letter from the head of the Bacteriological Department at LEO to R. G. Benedict at the Northern Regional Research Laboratory, U.S.A. stated that the company would be interested in receiving bacterial strains resistant to various known antibiotics. A draft list contained 30 species or types with the expectation that the final version would include around 50 species/types in addition to strains of rickettsia bacteria and viruses. In 1950 or 1951, several strains including two penicillin-resistant strains of *Staphylococcus aureus* and *Streptococcus pyogenes* were ordered from the National Collection of Type Cultures in United Kingdom. and the American Type Culture Collection as well as from the State Serum Institute (Supplementary Table S1)^27^. This strategy resembled the research leading to the discovery of neomycin in 1948/49 by the Waksman laboratory and the research later done by Bayer (Waksman and Lechevalier, 1949; Gradmann, 2016). Antibiotic-resistant strains in the target panel would both work as a dereplication technique to discard known antibiotics from the search process and as a means to find new useful antibiotics toward otherwise resistant target strains. The application of antibiotics for veterinary use, as feed supplements or as anti-cancer or anti-viral drugs presented additional objectives for the screening program^28^.

The program ran from 1949 to 1953, briefly revived from 1955 to 1956 (Figures 2, 3). Screening for antibiotic-producing cultures is only documented for the first period and work 1955–1956 most likely involved compounds from cultures isolated previously. Cultures of actinomycetes and in particular the genus *Streptomyces* were the major source of new natural product antibiotics during the 1940s and 1950s (Figure 1). These compounds also constituted the focus of LEO’s program. Soil is the main reservoir and the company outsourced the screening to an external expert in soil microorganisms, Hans Laurits Jensen at the State Laboratory of Plant Culture^29^. Most likely, H. L. Jensen was involved due to “the problem of proper identification (of antibiotic-producing *Streptomyces* cultures, my remark) is of considerable importance to investigators who apply for process or product patents” (Benedict, 1953). Indeed, as described for the American company Pfizer, “each species was investigated and registered by botanists, whose scientific discipline included the study of soil microorganisms” (Daemmrich, 2009).

The screening by Jensen used target bacteria with low pathogenic potential. The biological control laboratory at LEO also conducted initial screenings using *B. subtilis*, *E. coli*, *Sarcina* sp., *S. aureus*, and the fungus *C. albicans* as target cultures. Finally, the bacteriological research laboratory used a target panel of 10–15 pathogenic microorganisms. Tests were done on agar media at Jensen’s laboratory and in liquid media at the bacteriological department at LEO leading to different results. For this reason, LEO retested isolates for inhibition of bacterial pathogens and the bacteriological department switched to agar media to determine antimicrobial activity. However, many target microorganisms demanded specific growth conditions and could not be cultured together on agar plates. Further, safety concerns limited the extent of work on pathogenic cultures. This situation resulted already in 1951 in a bottleneck, as it was not possible to test weekly three antibiotic-producing cultures on 20 different media as initially suggested. This limited the number of possible target strains. Criteria for their selection included an important pathogenic role, easy to culture and not too dangerous to work with. If a sample gave a positive result for a selected bacterium, other species would be tested^30^. This made it important that target strains used for screenings were representative of the clinical microorganisms of interest. This experience was not unique to LEO, and a contemporary “lesson learned …(was) … that the substances one finds using a given screening method are not necessarily most effective against the target of the screen” (Lechevalier, 1980). The aim of finding the best target strain was a forerunner to standardize antibiotic testing although the major push in that regard came from the determination of antibiotic resistance (Gradmann, 2013).

Perhaps due to this bottleneck the company limited further work in 1952 to compounds already selected. One or two chemists from the hormone factory at LEO worked up to 1956 on 32 antibiotics that had passed the initial tests with four compounds analyzed in 1950, three in 1951, 13 in 1952–1953 and 12 in 1955–1956^31^. The chemists, at the same time also worked on heparin and adrenocorticotropic hormone (Schrøder et al., 2008)^32^. This illustrates the expertise at the company to characterize biological compounds, a line of work that differed from the synthetic chemistry done at the chemical research laboratory. Analyses included determinations of melting point, optical rotation, UV absorption, toxicity, antimicrobial spectrum and animal therapy experiments (Supplementary Table S2)^33^. In nine cases, the compounds were compared to known antibiotics by chromatographic analysis, a method commonly used at the time for this purpose (McGraw, 1976). At the end, all compounds were shelved, typically due to they were either known already, exhibited toxicity or some target organisms were resistant, all well-known obstacles in antibiotic screening programs.

The natural product antibiotic program involved several laboratories and departments within the company. The bacteriological department tested the inhibitory activity of antibiotic-producing cultures toward pathogenic target bacteria. The research laboratory at the penicillin factory optimized conditions for antibiotic production by testing different media, mutagenesis of producer strain or adding antibiotic precursors to the growth medium^34^. Chemists at the hormone factory purified and performed physicochemical analyses of antibiotics. Finally, the biological control laboratory at the penicillin factory carried out testing inhibitory activity toward mostly non-pathogenic bacteria and the biological department was involved in toxicity assays^35^. In conclusion, the company expertise included microbiological and biotechnological aspects, purification of organic compounds and competences in analytic and synthetic chemistry. This situation differed from the one described for Bayer where what was described as Bayer’s drug development department did not employ a single microbiologist in the 1950s (Gradmann, 2016).

## The Case for Serendipity: Discovery of Fusidic Acid in 1960

The empirical nature of natural product antibiotic search programs in the late 1940s and early 1950s is illustrated by this example: “It was a silly-simple project: collect soil samples, plate them out, isolate actinomycetes, test them for antibiotic activity against non-pathogenic strains of mycobacteria and hope you find something that will be active against the pathogenic strains” [Lechevalier (1980), recalling his research in the late 1940s in Salman Waksman’s laboratory]. Further, the high probability of rediscovering known compounds made large search programs necessary. Actinomycetes cultures producing the most commonly encountered antibiotic, streptothricin are found with a frequency of around 10% in nature, a number similar to the frequency of 12.5% of microorganisms with antimicrobial activity found by Eli Lilly from 1949 to 1959 (Table 3; Baltz, 2007). Actinomycetes cultures producing other antibiotics are found with lower frequencies and correspondingly larger screenings are necessary for their discovery (Baltz, 2007,2008). For Eli Lilly the overall number of strains screened over three decades amounted to 1,000,000 isolates and for Abbott laboratories about 400,000 isolated by the mid-1990s (Katz and Baltz, 2016). The number of isolates with antimicrobial activity obtained by LEO was nearly a 100-fold lower, compared to Eli Lilly (Table 3). There is no information on the number of environmental (soil) samples examined by LEO and the collaborator at the State Laboratory of Plant Culture but it most certainly was much lower than the 10,000 samples examined by Eli Lilly 1949–1959 and 100,000 samples examined by Pfizer in the late 1940s (Kahn, 1975; Greenwood, 2008; Daemmrich, 2009).

**TABLE 3:** Comparison of natural product antibiotic screenings by Eli Lilly and LEO 1949–1959^a^.

**Number of**	**Eli Lilly**	**LEO**
Soil samples	10,000	No data
Microorganisms examined	200,000	No data
Microorganisms with antimicrobial activity	25,000	300
Number of compounds analyzed	300	32
Number of compounds reaching the market	3	0

*^*a*^Data for Eli Lilly from Kahn (1975), and for LEO from this study.*

The outcome of the screenings by Pfizer and Eli Lilly was just one (oxytetracycline) and three (two of them erythromycin and vancomycin) antibiotics on the market and it is not surprising that LEOs limited efforts in the first half of the 1950s led to nothing. If the screening at LEO is compared with the work by two other small research units at the time, the ETH laboratory in Switzerland and the laboratory run by Salman Waksman at Rutgers University, the negative outcomes for LEO and the ETH were similar but opposed to the Waksman laboratory. The latter, however, initiated their work much earlier resulting in discoveries of several actinomycetes antibiotics, four of which found practical applications: Actinomycin (cancer), streptomycin (tuberculosis), neomycin (e.g., tuberculosis), and candicidin (anti-fungal). In all three cases actinomycetes antibiotics constituted a major focus but most of the low hanging fruits were gone by the time LEO and ETH initiated their work (Ettlinger, 1980; Lechevalier, 1980).

It is of interest that regarding natural product antibiotics LEO looked during the early 1950s for opportunities when possible. In one example, the company participated in an investigation that was a forerunner to the open innovation concept; a concept that recently had become popular among pharmaceutical companies including LEO Pharma (Nilsson and Felding, 2015). A confidential letter from 1951 describes the potential collaboration between the company and a medical doctor who had found an actinomycetes culture producing an antibiotic with no toxicity but with relatively strong *in vivo* inhibitory activity toward *Mycobacterium tuberculosis* using guinea pigs as an experimental model. The compound had not been isolated and therefore its chemical composition and structure was unknown. There were indications that it did not resemble known antibiotics used chemotherapeutically at the time. The company would aim at reproducing these findings and in case of an affirmative result, would conduct further investigations to explore possibilities for manufacturing the antibiotic in quantities for the use of chemotherapeutic treatment of human tuberculosis patients. In case of a positive outcome, the doctor was interested in LEO producing the compound on basis of a license^36^. Most likely, results did not support this prospect and no other documents in the archive refer to this case.

Another example of the search for the potential chance discovery was the testing of an antibiotic-producing culture accidentally obtained from the lab environment, however again without result^37^.

Success finally arrived with the discovery of fusidic acid in 1960, a serendipitous result of the work on semisynthetic β-lactams around this time (Rolinson, 1998; Kingston, 2000; Bud, 2007; Greenwood, 2008). This approach used mold-derived penicillin-degrading enzymes to obtain a penicillin core compound, 6-aminopenicillanic acid as a basic building block. As the scientists went through the catalog on mold cultures from a Dutch collection, they found a *Fusidium coccineum* (now *Acremonium fusidioides*) strain, an organism they had not heard about before. They decided to “very unscientifically to buy it – just because of the similarity of the name with *Fusarium*” – organisms they already used (quotation from Gotfredsen, 1997; the event is also described by Greenwood, 2008). Testing the strain for antibiotic production resulted in the discovery of fusidic acid, an anti-staphylococcal compound that has remained an important source of revenue for LEO (Schrøder, 2005; Schrøder et al., 2008; Fernandes, 2016). The discovery was timely due to contemporary issues in hospitals with antibiotic-resistant *S. aureus* (Schrøder et al., 2008).

It is instructive that LEO’s first real successful hit came when they examined the antibiotic potential of a genus of molds not tested previously, although related compounds, helvolic acid and cephalosporin P1 produced by other genera of molds were discovered much earlier, in 1943 and 1951 (Ritchie et al., 1951; Chain et al., 1953; Burton et al., 1956). Searching for new antibiotics among taxonomic groups of poorly characterized microorganisms remains one of the strategies for screenings programs today, with the discovery of teixobactin as one of many examples (Peláez, 2006; Aminov, 2010, 2017; Lewis, 2013, 2015; Genilloud, 2014; Ling et al., 2015). Serendipity did, however not guarantee any follow-up discoveries, and further searches by LEO for antibiotic-producing molds were not successful, as described by a scientist that took part in the work (A. Kjøller, personal communication). Since the strain that produced fusidic acid was isolated from monkey excrement in Japan, similar excrements from the local zoological garden in Copenhagen were a source of investigation in addition to soil samples from various locations in Denmark and abroad. Within a couple of years testing included thousands of strains but this led only to compounds that were either known already or too toxic. Actinomycetes antibiotics did not constitute a focus for this screening program. LEO continued work on antimicrobial compounds in the late 1960s and the 1970s and reported a few novel natural product compounds from cultures of *Streptomyces* or molds but no further R&D appear to have taken place (Gotfredsen and Vangedal, 1965; Von Daehne et al., 1969; Gotfredsen et al., 1970). The company eventually lost interest in antibiotics during the 1980s although fusidic acid is still manufactured today.

## Conclusion

This study explores the variety of strategies employed during the 1940s and 1950s for finding new natural, semisynthetic and synthetic antibiotic compounds. It is of interest, that LEO was a pharmaceutical company of relatively small size. Further, the owner from 1940 and until 1986, Knud Abildgaard aimed at rapidly securing marketable results from research. This offered less opportunity for larger strategic efforts, as opposed to numerous smaller projects. Although Abildgaard did not have a pharmaceutical or chemical background, he had, however, obtained an extensive amount of pharmaceutical knowledge (Schrøder, 2005; Schrøder et al., 2008). Taken together, these circumstances allowed LEO to maintain flexibility in strategies for R&D of antimicrobial compounds.

Research on synthetic antibiotics and salts and esters of penicillin benefited from the collaboration with K. A. Jensen as he tested antimicrobial activities, organized clinical trials and gave essential input as a consultant. He and other consultants also participated in regular meetings with the management of LEO. These meetings concluded by dinners that also served as incubators for professional interactions and even friendship and thereby fulfilling a role as a basis of knowledge and insights that served LEO well in their research (Jensen, 1971). The importance of this type of “privileged collaborative relations with clinical researchers” agrees well with the conclusions reached for Rhône-Poulenc’s research on anti-cancer drugs during the 1970s (Quirke, 2014).

Interestingly, the work on natural product antibiotics involved a collaborator, H. A. Jensen serving only a supportive role. This situation was not too different from the research that led to the discovery of chloramphenicol. “In 1943, no doubt alert to the work that Waksman was doing at Rutgers, but not yet wishing to commit company resources to an uncertain field of study, Parke, Davis and Co. in Detroit provided the funds for Paul Burkholder, Eaton professor of botany at Yale to screen samples of soil for micro-organisms producing substances with antibiotic activity” (Greenwood, 2008, p. 219). Similarly, LEO took a careful approach as at least the chemical analyses of these compounds constituted a relatively limited amount of overall research time (Table 2 and Figure 2).

In overall, the work by LEO came some way to meet the challenges at the time for finding new antibiotics. These included a need to be more precise in targeting the pathogens. This was the objective with the screening for antimicrobial compounds toward *M. tuberculosis*, and the search for compounds with a localized activity, e.g., an improved lung affinity. Further, the search included compounds with effect on antibiotic-resistant targets and/or on virulence rather than survival. In addition, the discovery of fusidic acid was a reward for testing microorganisms not previously well explored. Finally, there was an example of open innovation, a management mechanism that supports discoveries by searching outside the search parameters set by the company. All of these approaches are also in one way or another in play in the antibiotic discovery process today (Peláez, 2006; Baltz, 2008; Aminov, 2010, 2017; Rasko and Sperandio, 2010; Lewis, 2013, 2015; Genilloud, 2014).

## Author Contributions

JL designed the study, performed the research, and wrote the manuscript.

## Conflict of Interest

The author declares that the research was conducted in the absence of any commercial or financial relationships that could be construed as a potential conflict of interest.

## Footnotes

1 Report to management committee from Chemical Research Laboratory, December 1st, 1953.

2 Note 1, monthly time allocation on subjects the chemists in the chemical research laboratory worked on from 1947 to 1959 and an overview of research programs from March 30th, 1953.

3 See note 1.

4 Report from May 16th, 1952.

5 Documented by several letters between LEO, medical doctors and K. A. Jensen.

6 Meeting summary September 7th, 1954.

7 Document March 30th, 1953.

8 Remarks regarding research on tuberculosis, Chemical Research Laboratory, LEO, January 3rd, 1949.

9 See note 8. The object-glass method was mentioned in a discussion January 20th, 1954.

10 Overview of work in the Chemical Research Laboratory, November 13th, 1950.

11 Meeting summary with consultants September 5th, 1956.

12 Note 1, report, November 20th, 1950 and meeting summary January 3rd, 1949. Information on sulfones (e.g., derivatives of 4,4′-diaminodifenylsulfone) and sulfonamides (e.g., triphenylmethane sulfonamide) in letters June 9th, 1944 and June 19th, 1944. Work on thiosemicarbazones (concerning thiacetazone) is illustrated by laboratory reports, e.g., for the period between January 15th and April 15th, 1951.

13 Meeting summaries October 9th 1954, February 14th, 1955, June 12th, 1957 and August 15th, 1957.

14 Meeting summary February 10th, 1955.

15 See note 1.

16 Meeting summaries July 22nd, 1954 and September 7th, 1954.

17 Information on irritating effect is from meeting summaries January 10th 1955 and June 12th, 1957.

18 See note 2.

19 See note 2.

20 Note January 13th1942 on the application of sulfa drugs. Descriptions of specific compounds were found in letters and reports between K. A. Jensen and LEO June 9th, 1942: June 18th, 1942; September 30th, 1942; September 20th, 1943; October 10th, 1943; October 16th, 1943; November 19th, 1943; December 6th, 1943; December 12th, 1943; January 20th, 1944; February 29th, 1944; May 15th, 1944; June 6th, 1944; August 14th, 1944, September 18th, 1944.

21 Laboratory book 2, October 18th, 1951 to January 4th, 1956. See also note 1.

22 Report on work on new chemotherapeutics July 1st-July 31st, 1944.

23 Documented by numerous laboratory and meeting reports.

24 See note 1.

25 Overview October 16th, 1952 on research tasks of the factory.

26 Undated proposal for routine testing of substrate samples on pathogenic bacteria, including 30 (unspecified) species and strains “that are not satisfactorily treatable with penicillin, streptomycin or sulfonamides and which plays an important role in human or veterinary pathology.”

27 Letter to Benedict, April 20th, 1949. Letters from LEO to culture collections, see Supplementary Table S1.

28 Feed supplement: Letter from LEO to a Danish company November 29th, 1950. Cancer treatment: Note November 18th, 1954 on Actinomycin C and a report on microbial chemotherapy, September 1st, 1953. Anti-viral effects: Procedure March 6th, 1954. Veterinary applications: Numerous documents.

29 Report November 18th 1950 on experimental activity in pilot plant of the penicillin factory. Note December 6th 1951 on antibiotic research from Bacteriological Department. Note December 7th 1951 describing current procedure for antibiotic screening and suggestion for changes of procedure. Meeting summary December 13th 1951 on guidelines regarding antibiotic research.

30 Meeting summary August 27th, 1951. Changed procedure for selection of antibiotic-producing cultures, December 5th, 1951. Note on collaboration between Biological Research Laboratory and Bacteriological Research Laboratory March 6th, 1952. Suggestion August 8th, 1952 for changes in guidelines regarding antibiotic research. LEO also analyzed an unknown number of their own soil samples (Undated document). See also note 26.

31 Laboratory book 1 October 12th, 1951 to November 26th, 1952. Laboratory book 2, see note 21. Note December 6th, 1951.

32 Also documented by the monthly time allocation on subjects the chemists were working on.

33 Meeting summary on comparing an unknown antibiotic with patulin, October 1st, 1951. Description December 20th, 1952 of testing an antibiotic. Note December 6th, 1951 on antibiotic research from Bacteriological Department. Overview October 20th, 1952 on test results for six new antibiotics. Note December 20th, 1952 on purification of an antibiotic and testing antimicrobial spectrum. Laboratory book 1 and Laboratory book 2. See also Supplementary Table S2.

34 Report November 18th, 1950 on experimental activity in the pilot plant of the penicillin factory.

35 Note March 6th, 1952 regarding standardizing the target bacterial panel. Several documents refer to bacteriological and toxicity assays done by the Biological Department.

36 Meeting summary December 7th, 1951.

37 Document January 24th, 1952.

## References

[B1] Abouelhassan Y. Garrison A. T. Yang H. Chávez-Riveros A. Burch G. M. Huigens R. W. (2019). Recent progress in natural-product-inspired programs aimed to address antibiotic resistance and tolerance. *J. Med. Chem.* 62 7618–7642. 10.1021/acs.jmedchem.9b00370 30951303 PMC6742553

[B2] Aminov R. (2017). History of antimicrobial drug discovery: major classes and health impact. *Biochem. Pharmacol.* 133 4–19. 10.1016/j.bcp.2016.10.001 27720719

[B3] Aminov R. I. (2010). A brief history of the antibiotic era: lessons learned and challenges for the future. *Front. Microbiol.* 1:134 10.3389/fmib.2010.00134 PMC3109405 21687759

[B4] Anon. (1958). *US Federal Trade Commission, Economic Report on Antibiotics Manufacture.* Washington, DC: U.S. Government Printing Office.

[B5] Baltz R. H. (2007). Antimicrobials from actinomycetes: back to the future. *Microbe* 2 125–131.

[B6] Baltz R. H. (2008). Renaissance in antibacterial discovery from actinomycetes. *Curr. Opin. Pharmacol.* 8 557–563. 18524678 10.1016/j.coph.2008.04.008

[B7] Benedict R. G. (1953). Antibiotics produced by actinomycetes. *Botan. Rev.* 15 229–314.

[B8] Bud R. (2007). *Penicillin. Triumph and Tragedy.* Oxford: Oxford University Press.

[B9] Burton H. S. Abraham E. P. Cardwell H. M. E. (1956). Cephalosporin P_1_ and helvolic acid. *Biochem. J.* 62 171–176. 13293169 10.1042/bj0620171 PMC1274528

[B10] Chain E. Florey H. W. Jennings M. A. Williams T. I. (1953). Helvolic acid, an antibiotic produced by *Aspergillus fumigatus*. mut. helvola Yuill. *Brit. J. Exp. Pathol.* 24 108–119.

[B11] Cozzoli D. (2014). Penicillin and the European response to post-war American hegemony: the case of LEO-penicillin. *Hist. Technol.* 30 83–103.

[B12] Daemmrich A. (2009). Synthesis by microbes or chemists? Pharmaceutical research and manufacturing in the antibiotic era. *Hist. Technol.* 25 237–256.

[B13] Espersen E. (1949). Morphological studies of the normal growth of a human tubercle strain, and the effects of some antibacterial substances on same. *Acta Pathol. Scand.* 26 178–204. 10.1111/j.1699-0463.1949.tb03154.x 18117527

[B14] Ettlinger L. (1980). “Wartime research on penicillin in Switzerland and antibiotic screening,” in *The History of Antibiotics. A Symposium*, ed. Parascandola J. (Madison, WI: American Institute of the History of Pharmacy), 57–67.

[B15] Fernandes P. (2016). Fusidic acid: a bacterial elongation factor inhibitor for the oral treatment of acute and chronic staphylococcal infections. *Cold Spring Harb. Perspect. Med.* 6:a025437. 10.1101/cshperspect.a025437 26729758 PMC4691801

[B16] Fox G. J. Schaaf H. S. Mandalakas A. Chiappini E. Zumla A. Marais B. J. (2017). Preventing the spread of multidrug-resistant tuberculosis and protecting contacts of infectious cases. *Clin. Microbiol. Infect.* 23 147–153. 10.1016/j.cmi.2016.08.024 27592087 PMC11849722

[B17] Frederiksen E. Jensen K. A. Morch P. Tybring L. (1957). Tebamin: a derivative of p-aminosalicylic acid; blood levels, absorption and excretion following oral administration. *Acta Pharmacol. Toxicol.* 14 58–66. 10.1111/j.1600-0773.1957.tb01142.x 13469430

[B18] Genilloud O. (2014). The re-emerging role of microbial natural products in antibiotic discovery. *Ant. Leeuw.* 106 173–178. 10.1007/s10482-014-0204-6 24923558

[B19] Gotfredsen W. O. (1991). Penicillinet 50 år. En beretning om en af medicinhistoriens store opdagelser. *Løvens Litter. Inform.* 23 149–165.

[B20] Gotfredsen W. O. (1997). *Om Opdagelsen og Udviklingen af Fucidin.* Ballerup: Løvens Kemiske Fabrik.

[B21] Gotfredsen W. O. Vangedal S. (1965). Trichodermin, a new sesquiterpene antibiotic. *Acta Chem. Scand.* 19 1088–1102. 10.3891/acta.chem.scand.19-1088 5850137

[B22] Gotfredsen W. O. Vangedal S. Thomas D. W. (1970). Cycloheptamycin, a new peptide antibiotic. Structure determination by mass spectrometry. *Tetrahedron* 26 4931–4946. 10.1016/s0040-4020(01)93145-x 5500058

[B23] Gotfredsen W. O. (1999a). *Om Opdagelsen og Udviklingen af Pondocillin.* Ballerup: Løvens Kemiske Fabrik.

[B24] Gotfredsen W. O. (1999b). *Om Opdagelsen og Udviklingen af Sultamicillin.* Ballerup: Løvens Kemiske Fabrik.

[B25] Gradmann C. (2013). Sensitive matters: the world health organisation and antibiotic resistance testing, 1945-1975. *Soc. Hist. Med.* 26 555–574.

[B26] Gradmann C. (2016). Re-inventing infectious disease: antibiotic resistance and drug development at the Bayer company 1945-1980. *Med. Hist.* 60 155–180. 10.1017/mdh.2016.2 26971595 PMC4847408

[B27] Greenwood D. (2008). *Antimicrobial Drugs. Chronicle of a Twentieth Century Medical Triumph.* Oxford: Oxford University Press.

[B28] Høiby N. (2000). “Der er to slags forskere, de positive og de negative,” in *Min Bedste Lærer*, ed. Riis J. (Copenhagen: Gyldendalske Boghandel, Nordisk Forlag A/S Copenhagen), 116–128.

[B29] Høiby N. (2016). Kai Adolf Jensen, professor i almindelig patologi. *Bibliotek for Læger* 208 344–361.

[B30] Jain S. K. Lamichhane G. Nimmagadda S. Pomper M. G. Bishai W. R. (2008). Antibiotic treatment of tuberculosis: old problems, new solutions. *Microbe* 3 285–292.

[B31] Jensen K. (2002). *Bekæmpelse af Infektionssygdomme – Statens Serum Institut 1902-2002.* Copenhagen: Nyt Nordisk Forlag Arnold Busck.

[B32] Jensen K. A. (1971). Fabrikejer Knud Abildgaard 70 år. *Medicinsk Forum* 23/24 16–19.

[B33] Jensen K. A. Dragsted P. J. Kiaer I. Nielsen E. J. Frederiksen E. (1951). Leocillin (Benzylpenicillin-β-diethylaminoethylesterhydrochloride). *Acta Pathol. Microbiol. Scand.* 28 407–414. 14856746

[B34] Kahn E. J. Jr. (1975). *All in a Century. The First 100 Years of Eli Lilly and Company.* Indianapolis, IN: Eli Lilly and Company.

[B35] Kane J. H. Finlay A. C. Sobin B. A. (1950). Antimicrobial agents from natural sources. *Ann. N.Y. Acad. Sci.* 53 226–228. 14783329 10.1111/j.1749-6632.1950.tb42153.x

[B36] Katz L. Baltz R. H. (2016). Natural product discovery: past, present and future. *J. Ind. Microbiol. Biotechnol.* 43 155–176. 26739136 10.1007/s10295-015-1723-5

[B37] Kavanagh F. Hervey A. Robbins W. J. (1951). Antibiotic substances from Basidiomycetes: VII. *Pleurotus mutilus* (FR.) sacc. and *Pleurotus passeckerianus* Pilat. *Proc. Natl. Acad. Sci. U.S.A.* 37 570–574. 10.1073/pnas.37.9.570 16589015 PMC1063423

[B38] Keshavjee S. Farmer P. E. (2012). Tuberculosis, drug resistance, and the history of modern medicine. *N. Engl. J. Med.* 367 931–936. 10.1056/NEJMra1205429 22931261

[B39] Kingston W. (2000). Antibiotics, invention and innovation. *Res. Policy* 29 679–710.

[B40] Lechevalier H. A. (1980). “The search for antibiotics at Rutgers university,” in *The History of Antibiotics. A Symposium*, ed. Parascandola J. (Madison, WI: American Institute of the History of Pharmacy), 113–123.

[B41] Lewis K. (2013). Platforms for antibiotic discovery. *Nat. Rev. Drug Dis.* 12 371–387. 10.1038/nrd3975 23629505

[B42] Lewis K. (2015). Challenges of antibiotic discovery. *Microbe Wash. D. C.* 10 363–369.

[B43] Ling L. L. Schneider T. Peoples A. J. Spoering A. L. Engels I. Conlon B. P. (2015). A new antibiotic kills pathogens without detectable resistance. *Nature* 517 455–459. 10.1038/nature14303 25561178 PMC7414797

[B44] Loldrup H.-O. (2014). *Dansk Medicin. Historien om de Danske Medicinfabrikker.* Copenhagen: Loldrups forlag.

[B45] McGraw D. J. (1976). *The Antibiotic Discovery Era (1940-1960): Vancomycin as an Example of the Era.* Ph.D. thesis. Oregon State University, U.S.A, Eugene, OR.

[B46] Nilsson N. Felding J. (2015). Open innovation platform to boost pharmaceutical collaborations: evaluating external compounds for desired biological activity. *Future Med. Chem.* 7 1853–1859. 10.4155/fmc.15.122 26393392

[B47] Peláez F. (2006). The historical delivery of antibiotics from microbial natural products – Can history repeat? *Biochem. Pharmacol.* 71 981–990. 10.1016/j.bcp.2005.10.010 16290171

[B48] Quinn R. (2009). *Broader Spectrum: A History of Antibiotic R&D.* Ph.D. Dissertation. University of Urbana-Champaign, Urbana, IL.

[B49] Quirke V. (2014). Targeting the American market for medicines, ca. 1950s-1970s: ICI and Rhône-Poulenc compared. *Bull. Hist. Med.* 88 654–696. 10.1353/bhm.2014.0075 25557515 PMC4335572

[B50] Raper K. B. (1952). A decade of antibiotics in America. *Mycologia* 44 1–59.

[B51] Rasko D. A. Sperandio V. (2010). Anti-virulence strategies to combat bacteria-mediated disease. *Nat. Rev. Drug discov.* 9 117–128. 10.1038/nrd3013 20081869

[B52] Ritchie A. C. Smith N. Florey H. W. (1951). Some biological properties of cephalosporin P_1_. *Br. J. Pharmacol.* 6:430. 10.1111/j.1476-5381.1951.tb00653.x PMC1509140 14878978

[B53] Rolinson G. N. (1998). Forty years of β-lactam research. *J. Antimicrob. Chemother*. 41 589–603. 9687097 10.1093/jac/41.6.589

[B54] Schrøder P. (2005). Fra løveapotekets kemiske fabrik til LEO Pharma A/S. *Theriaca* 36 27–118. 17144611

[B55] Schrøder P. Kirkegaard H. Holmstad D. (2008). *Leo Pharma 1908-2008.* Ballerup: LEO Pharma.

[B56] Silver L. L. (2011). Challenges of antibacterial discovery. *Clin. Microbiol Rev.* 24 71–109. 21233508 10.1128/CMR.00030-10 PMC3021209

[B57] Spellberg B. (2014). The future of antibiotics. *Critical Care* 18:228. 10.1186/cc13948 PMC4075146 25043962

[B58] Tanner L. Denti P. Wiesner L. Warner D. F. (2018). Drug permeation and metabolism in *Mycobacterium tuberculosis*: prioritising local exposure as essential criterion in new TB drug development. *IUBMB Life* 70 926–937. 10.1002/iub.1866 29934964 PMC6129860

[B59] Temin P. (1979). Technology, regulation, and market structure in the modern pharmaceutical industry. *Bell J. Econ.* 10 429–446.

[B60] Theuretzbacher U. Piddock L. J. V. (2019). Non-traditional antibacterial therapeutic options and challenges. *Cell Host Micr.* 26 61–72. 10.1016/j.chom.2019.06.004 31295426

[B61] Tjørnelund H. (2016). *Da det Frelsende mug Kom til København. En Videnskabs og Teknologihistorisk Afhandling om Udviklingen af den Første Danske Penicillin Fra 1942 Til 1959.* Ph.D. Thesis, Roskilde University, Roskilde.

[B62] Tørning K. Jensen K. A. Kiaer I. (1958). Clinical Studies on Tebamin. *Acta Tuberc. Scand.* 35 87–100.

[B63] Umezawa H. Kondo S. Meda K. Okami Y. Okada T. Takeda K. (1967). *Index of Antibiotics From Actinomycetes.* State College, PA: University Park Press.

[B64] Von Daehne W. Gotfredsen W. O. Tybring L. Schaumburg K. (1969). New antibiotics containing the 1,2-dithiolo[4,3-b]pyrrole ring system. *J. Antibio.* 22 233–236. 10.7164/antibiotics.22.233 5811398

[B65] Waksman S. A. Lechevalier H. A. (1949). Neomycin, a new antibiotic active against streptomycin-resistant bacteria, including tuberculosis organisms. *Science* 109 305–307. 10.1126/science.109.2830.305 17782716

[B66] Waksman S. A. Lechevalier H. A. (1953). *Actinomycetes and Their Antibiotics.* Baltimore: The Williams & Wilkins Company.

[B67] Wright G. D. (2014). Something old, something new: revisiting natural products in antibiotic drug discovery. *Can. J. Microbiol.* 60 147–154. 10.1139/cjm-2014-0063 24588388

[B68] Wright P. M. Seiple I. B. Myers A. G. (2014). The evolving role of chemical synthesis in antibacterial drug discovery. *Angew. Chem. Int. Ed. Eng.* 53 8840–8869. 10.1002/anie.201310843 24990531 PMC4536949

