# Supplementary material for: The Diverse Search for Synthetic, Semisynthetic and Natural Product Antibiotics From the 1940s and Up to 1960 Exemplified by a Small Pharmaceutical Player
Source: Front Microbiol. 2020 Jun 12;11:976. doi: 10.3389/fmicb.2020.00976 (PMC7303287; doi:10.3389/fmicb.2020.00976)
Supplement: Supplementary file 1 [file Data_Sheet_1.docx]

**Appendix 1: Data on discovery rates for actinomycetes antibiotics 1940-1965**

The main set of data were retrieved from Umeza et al. 1967, Chapter 2 and 3. Compounds with no information on antimicrobial activity or with activity only towards viruses, bacteriophages, tumor cells, fungi or yeasts were not included. Compounds were listed according to the publishing year of the first scientific report or patent. Several compounds, including complex components were indicated as identical and accepted as such if there were only minor differences in relation to formulas, chemical compositions, melting points, optical rotations, molecular weights and infrared and UV spectra. In such cases, the initial discovery was included. Some compounds listed as different components under the same entry were included as separate entities. Information in Umeza et al. 1967 sometimes disagree with other data. Thus, foromacidins have been described as identical with spiramycins (Ettlinger 1980) whereas Umeza, et al. 1967 gave deviating molecular weights and describe these compounds only as similar. In such cases, I followed decisions outlined in Umeza et al. 1967.

Many antibiotics are produced as complexes of slightly different components by the same culture. In some cases, such components were listed as separate antibiotics whereas in other cases they were listed under the same antibiotic entry. In the latter case, the complex components were included as separate entities except if there was a lack of information on components or some components were similar to other antibiotics discovered previously.

Additional data were retrieved from Waksman and Lechevalier, 1953 in a similar manner and again only including compounds that showed antibacterial activity.
