# Supplementary material for: The Diverse Search for Synthetic, Semisynthetic and Natural Product Antibiotics From the 1940s and Up to 1960 Exemplified by a Small Pharmaceutical Player
Source: Front Microbiol. 2020 Jun 12;11:976. doi: 10.3389/fmicb.2020.00976 (PMC7303287; doi:10.3389/fmicb.2020.00976)
Supplement: Supplementary file 2 [file Table_1.pdf]

Suppl. Table 1. Bacterial strains ordered from culture collections.

Letter to:	Name indicated in letter	Current name
American Type Culture Collection 28/11 1950	*Bacillus proteus*	*Proteus vulgaris*
American Type Culture Collection 15/3 1951	*Vibrio metchnikovi*<br>*Mycobacterium lepraemurium*	*V. metschnikovii?*<br>*M. lepraemurium*
National Collection of Type Cultures, U.K. 9/10 1951	*Corynebacterium pyogenes*, bovine strain	*Trueperella pyogenes*
	Corynebacterium pyogenes, porcine strain	*Trueperella pyogenes*
	Mycobacterium johnei	*M. paratuberculosis*
	Bacillus anthracis, avirulent strain	*B. anthracis*
	Bacillus anthracis, virulent strain	*B. anthracis*
	Clostridium botulinum, type A	*C. botulinum*
	Erysipelothrix rhusiopathiae	*E. rhusiopathiae*
	Fusiformis necrophorus	*Fusobacterium necrophorum*
	Bacterium rhinoscleromatis	*Klebsiella pneumoniae* subsp. rhinoscleromatis
	Salmonella Abortus-equi	*S.* Abortus-equi
	Salmonella Dublin	*S.* Dublin
	Salmonella Pullorum	*S.* Pullorum
	Vibrio fetus	*Campylobacter fetus*
	Staphylococcus aureus, pathogenic to rabbits	*S. aureus*
	Staphylococcus aureus, resistant to penicillin	*S. aureus*
	Streptococcus, Lancefield group A, resistant to penicillin	*S. pyogenes*
	Haemophilus bronchisepticus	*Bordetella bronchiseptica*
	Brucella melitensis	*B. melitensis*
	Brucella suis	*B. melitensis*
Internal letter 29/11 1951. Strain from State Serum Institute	*Streptococcus,* Grp. A.	*S. pyogenes*
