# Supplementary material for: The Diverse Search for Synthetic, Semisynthetic and Natural Product Antibiotics From the 1940s and Up to 1960 Exemplified by a Small Pharmaceutical Player
Source: Front Microbiol. 2020 Jun 12;11:976. doi: 10.3389/fmicb.2020.00976 (PMC7303287; doi:10.3389/fmicb.2020.00976)
Supplement: Supplementary file 3 [file Table_2.docx]

**Suppl. Table 2. Examples on characterization of natural product antibiotics^a^**

| Strain | Chemical analyses^b^ | | | UV  (mµ) | Toxi-city test  (LD_50_) | Inhibition of | | | | The-rapy  effect |
| --- | --- | --- | --- | --- | --- | --- | --- | --- | --- | --- |
|  | Mp^c^  (C^o^) | Rota-tion | MW^d^ |  |  | Gram pos. | Gram neg. | Acid-  Fast^e^ | Molds |  |
| MY-47 | 250 | -334^o^ | 1020 | 240, 445 | 3-7^f^ | +++ | + | +++ | - | ND^g^ |
| MY-51 | 250 | +109^o^ | ND | 237, 310 | 50^i^ | +++ | + | +++ | + | ND |
| P20 | 125-128 | -129^0^ | ND | NI^h^ | 100^f^ | ++ | ++ | ND | + | - |
| ØA-8 | ND | ND | ND | ND | High | + | + | ND | + | ND |
| MÆ-32 | ND | ND | ND | ND | ND | ++ | +++ | ND | - | ND |
| MZ-37 | ND | ND | ND | ND | ND | + | +++ | ND | + | -/+^j^ |

^a^Data from overview on tests results (October 20th 1952) and entries in Lab book 1 October 12^th^ 1951 to November 26^th^ 1952 and Lab book 2 October 18th 1951 to January 4th 1956.

^b^General purification approach: Medium with grown culture was mixed with solvent at a given pH value (e.g. neutral or weakly acidic), evaporated, dissolved in organic solvent, separated by chromatography (e.g. Al_2_O_3_ or silica gel); active fraction concentrated by evaporation, precipitated by e.g. (NH_4_)_2_SO_4_, alcohol, acetone or ether and finally re-crystallized. Stability tests regarding pH and heat were also conducted. Majority of compounds were stable within a pH range from 1 to 11 and many also retained activity after exposure to 100^o^C.

^c^Melting point

^d^Molecular weight

^e^*Mycobacterium*

^f^mg/kg

^g^Not done or no data (ongoing experiment)

^h^Not identified

^i^mg/kg subcutaneous

^j^Active against Friedland bacillus (*Klebsiella pneumoniae*) by parenteral but not peroral administration.
